# The Uncommon Impact of Common Environmental Details on Walking in Older Adults

**DOI:** 10.3390/ijerph14020190

**Published:** 2017-02-14

**Authors:** Katherine Brookfield, Catharine Ward Thompson, Iain Scott

**Affiliations:** Edinburgh College of Art, University of Edinburgh, Edinburgh EH3 9DF, UK; c.ward-thompson@ed.ac.uk (C.W.T.); iain.scott@ed.ac.uk (I.S.)

**Keywords:** walking, older adults, physical activity, environment

## Abstract

Walking is the most common form of physical activity amongst older adults. Older adults’ walking behaviors have been linked to objective and perceived neighborhood and street-level environmental attributes, such as pavement quality and mixed land uses. To help identify components of walkable environments, this paper examines some of these environmental attributes and explores their influence on this population’s walking behaviors. It draws on focus group and interview data collected from 22 purposively sampled older adults aged 60 years and over. These participants presented a range of functional and cognitive impairments including stroke and dementia. In line with past research, we detail how various everyday aspects of urban environments, such as steps, curbs and uneven pavements, can, in combination with person-related factors, complicate older adults’ outdoor mobility while others, such as handrails and benches, seem to support and even encourage movement. Importantly, we delineate the influence of perceptions on mobility choices. We found that, in some instances, it is the meanings and possibilities that older adults derive from aspects of the environment, such as street cameras and underpasses, rather than the aspects per se, which shape behavior. The implications for policy and practice are considered.

## 1. Introduction

Walking is a low-cost, accessible form of physical activity [1] that can bring important health benefits to all ages, including older adults [2], and help maintain functional independence in old age [3]. It is also the most common form of physical activity within the older population [4].

Various studies have investigated possible environmental correlates of walking in general populations of older adults. They have highlighted links between walking and mixed land uses [5], proximity to recreational uses [6], density [7], pedestrian infrastructure [8], perceptions of safety for walking [9], the quality of neighborhood open space and the quality of paths to such space [10,11]. However, a recent systematic review of studies into links between objective and perceived neighborhood and street-level environmental details and forms of physical activity, including walking, in this age group found few significant relationships [12]. Stressing, though, that this finding might merely be a function of certain methodological issues within this developing area of research, the review authors argued for studies to continue to explore possible links between the environment, walking, and other varieties of physical activity in seniors [12].

Environmental design and gerontology studies have identified a range of (usually) street-level factors as potential influences on older adults’ walking behaviors and experiences. Items highlighted include the condition, width and evenness of footways, pavement obstructions (such as cars parked on pavements), tactile paving, dropped curbs, handrails, benches, street greenery and the accessibility, safety and cleanliness of public toilets [13]. Further, research on environmental factors associated with falls and the fear of falling in older adults have identified environmental features, again usually at street-level, that require particular attention, such as path materials and obstructions [14]. Research relating the environment to people’s desired activities has emphasized the concept of “environmental supportiveness” [15,16] and shown how perceptions of the quality and attractiveness of outdoor environments, as well as their functional characteristics, influence whether older people will choose to go out and what they are prepared to do if they go out [17]. For example, tree-lined walks and seats *en route* to a destination constitute a supportive environment for many older people, one that makes it easy and enjoyable to use, while dog fouling or signs of vandalism may inhibit outdoor activity [10]. Stated preference techniques have also been used to help understand the relative importance of such environmental qualities in supporting or inhibiting older people’s visits to outdoor places, e.g., a local park [18,19]. Often, this collection of studies has tended to engage with relatively healthy and physically active older adults.

To further widen and deepen our understanding of the environment’s relationship to walking in older adults, this paper presents findings from a qualitative study on age-, stroke- and dementia-friendly environments that engaged with a diverse sample of adults aged 60 years and over [20]. The paper has a particular take on environmental supportiveness, informed by the Person-Environment (P-E) fit, or ecological, model which argues that interactions between the person and the environment structure how an individual adapts to, and ‘performs’ within, a given situation. The person is defined in terms of a set of competencies and the environment in terms of demands [21]. The environment encompasses the physical environment, personal environment, small group environment, suprapersonal environment and social or megasocial environment [22]. The personal factors of concern include an individual’s biological health, sensory and motor skills, and cognitive function [21]. Where there is poor alignment, or a poor fit, between these person-related and environmental factors, i.e., between competencies and demands, adaptation is low and performance impaired. Tasks may be difficult to complete, assistance may be required. As competences differ between individuals, within a given environment interactions between person-related and environmental factors will vary from person to person. The same environment will thus be experienced differently by different people [17]. Focusing on this issue, this paper aims to explore how outdoor environments are experienced by diverse older adults. Specifically, it investigates how neighborhood and street-level environmental factors interact with person-related factors to inform walking behaviors in assorted older adults.

## 2. Materials and Methods

We asked 22 purposively sampled older adults (see later), aged 60 years and over, to take part in a face-to-face, semi-structured interview and two focus groups. The interviews and focus groups explored participants’ perspectives on, and experiences within, diverse outdoor environments and the home. Ethical approval for the study was provided by the National Health Service (NHS) West of Scotland Research Ethics Committee (REC) 3 (REC Ref: 13/WS/0183) and a Research Ethics Committee at the authors’ institution.

The first focus group explored participants’ emotional responses to places and their preferences in relation to indoor and outdoor environments. The first author, assisted by the third author, acted as the focus group moderator. Focus groups have been employed in research with older people [23], stroke survivors [24,25] and people with dementia [26] while the advantages and limitations of the method are well documented [27].

The semi-structured interview [28], comprised three parts, and was conducted by the first author. The first part explored attitudes towards the home, including how individuals felt within the home, and activities completed at home. The second part explored place perception using photo-elicitation. Here, images are introduced into an interview to stimulate reflection and comment [29]. Picture-based methods can help people with dementia and communication difficulties express their views [30,31]. Six researcher-generated photographs showing different outdoor environments were introduced into the interview and the thoughts, attitudes and feelings they inspired were explored. Three photographs showed environments containing features that older adults are thought to appreciate (e.g., neighbours interacting), according to research conducted by the World Health Organization (WHO) [32], while three showed features older adults are thought to dislike (e.g., litter), again according to this research. The final part of the interview employed the Talking Mats^TM^ communication framework, a low-technology, picture-based communication framework developed to help individuals with communication difficulties express their views [31]. Further information on, and images of, this framework can be viewed at [33]. We used this framework with all of our participants to explore their views on the relative importance of various aspects of outdoor environments and the home. In this framework, pictures illustrate a topic—an activity, item or relationship, for example—and individuals indicate their attitudes towards this topic by placing it somewhere along a visual scale [31]. The visual scale we employed explored the concept of “importance” and ranged from “not important” to “important”. The pictures we used illustrated 17 features of the home and outdoor environments that have been identified by the WHO [32] as forming necessary components of an age-friendly home and city. Features were varied and included such items as kitchen facilities, bathroom facilities, greenspace and pavements.

The final focus group involved participants reviewing and discussing architectural propositions for age-, dementia- and stroke-friendly indoor and outdoor environments devised by a group of student architects. The students had developed their designs through reference to findings from the first focus group and the semi-structured interviews. The first and third author presented the propositions in layman’s terms, talking through architectural drawings and models created by the students.

The interviews and focus groups were audio-recorded and transcribed. Field notes were made within and immediately after each interview and focus group, capturing information on participant characteristics and group dynamics.

### 2.1. Participants and Recruitment

To understand an older person’s experiences within the built environment, we must provide opportunities for older adults to describe their experiences in their own words [34]. Lived or first-person experiences can provide a richness or depth of insight not possible in accounts collected from proxies while, pertinent to our study, multiple past studies have successfully collected data directly from older adults [23,35], stroke survivors [25,36] and people with dementia [26,37]. With this concern in mind, working within certain time and resource constraints [38], we sought to assemble a diverse sample [39]. Ultimately, we recruited to our study fifteen “healthy volunteers” (“healthy” in so far as these individuals were not recruited to present any particular diagnosed condition although, as will be seen, many subsequently reported diagnosed and general health complaints and conditions), five stroke survivors (patients discharged from hospital six months to two years previously), and two community-dwelling older adults with a confirmed diagnosis of dementia. The participants with dementia had an Addenbrooke’s Cognitive Examination-Revised (ACE-R) Total equal to or greater than 10, which excludes people with severe cognitive impairment. All participants had the capacity to consent. Ethical approval for our study required that the participants with dementia had an observer (such as a partner, family member or care-giver) present at the interview and focus groups. Gatekeepers and issues with poor health impacted the number of stroke survivors and people with dementia recruited to the study. A further individual attended the first focus group convened for the healthy volunteers but, being under the age of 60, they were subsequently excluded from the study.

The healthy volunteers were recruited through older people’s groups and community organizations. The stroke survivors were identified and contacted by clinicians who work with this population. The participants with dementia were recruited through the Scottish Dementia Clinical Research Network’s dementia research interest register.

Participants lived in a mix of urban and suburban areas within Edinburgh, the capital city of Scotland. Edinburgh is a densely populated city of 493,000 people [40] and features a wide variety of types of environment; from a medieval Old Town to the neo-classical C18th and C19th New Town and C20th apartment blocks and villas, and from tightly packed streets of tenement flats to low density suburban housing. Participants were drawn from a variety of these environments.

Participants were invited to take part in all phases of data collection—the first focus group, an interview and the second focus group. The goal was for participants to take part in all three phases. However, some individuals did not wish or were unable (due to other commitments) to participate in all phases. For the healthy volunteers, 12 participants took part in the first focus group, 14 took part in an interview and 11 took part in the final focus group. For the stroke survivors, four participants took part in the first focus group, four took part in an interview and three took part in the final focus group. For the people with dementia, two participants took part in an interview. No focus groups were organized for this set of participants owing to the low number of participants recruited and lack of interest in participating in focus groups. Separate focus groups were convened for the stroke survivors and the healthy volunteers.

### 2.2. Analysis

Short summaries of the interviews and focus groups were prepared by reference to the field notes. A preliminary thematic analysis [41] was performed on these summaries using an inductive approach informed by findings and theories from the environmental gerontology literature, including the P-E fit model. The summaries relating to the three groups of participants were analyzed separately to maximize opportunities to identify differences and similarities between, and consistencies within, each set. Themes identified included residential preferences, environment and physical activity, health, ageing and physical activity, environment and affect, important components of a home/neighborhood and activities/pastimes. A preliminary coding framework was assembled from these themes. This framework steered a line-by-line thematic analysis of the focus group and interview transcripts performed within NVivo^TM^ (QSR International, Doncaster, Australia) [42]. During this analysis, the coding framework was enriched through the addition of categories and codes identified within the raw data [39]. This inductive analysis, informed, as before, by findings and theories from the environmental gerontology literature, mainly uncovered additional layers and facets in existing themes, bringing depth and nuance to the findings.

## 3. Results

Our findings indicated that various everyday aspects of urban environments could, in combination with person-related factors, have a discernible impact on the outdoor mobility of diverse older adults. The impact was often greatest in those reporting physical, visual and/or cognitive impairments. Various environmental details were found to frustrate walking, forming, in a very real sense, physical barriers to movement; examples included steps, slippery surfaces, uneven pavements and curbs. Other items appeared to have the capacity to support and even encourage movement. Benches, for instance, provided a welcome resting point, helping to facilitate longer trips. Importantly, in a number of cases, we found that what mattered were the meanings and possibilities that older adults located within aspects of the environment. Negative perceptions of certain places, environmental features and times of the day could lead individuals to eschew some spatial and temporal settings. It was commented that these perceptions, and this avoidance behavior, had developed as participants had got older. These key findings are discussed below. Pseudonyms are provided for all participants to maintain anonymity.

### 3.1. Environmental Barriers

Various everyday components of urban environments could, in combination with person-related factors, such as a health condition, compound existing difficulties, introduce new complications and detract from an individual’s overall “walking experience”. Similar to much past research, problematic items tended to occur at street-level and included curbs, steps, uneven and slippery surfaces, cluttered pavements, inclines and poor street lighting. In settings where two or more of these items were present, difficulties could be further exacerbated with the complications arising from one item reinforced by complications arising from the second. The impact of these environmental details appeared most pronounced in individuals with mobility limitations. Amala, a healthy volunteer, discussed how curbs, in combination with arthritis in her hips, introduced difficulty and inconvenience into everyday journeys:

“*When I’m walking now I have to walk right to the end of the street where the pavement sort of drops down and then walk across and up and sometimes, if I’m getting off the bus, I have to walk a distance to find that space because climbing up and down off the pavement really hurts my hips.*”

Some participants had developed coping strategies to contend with the obstacles they encountered when going outdoors. James, recruited as a healthy volunteer but reporting mobility limitations that sometimes required the use of a mobility scooter, restricted local trips to a particular set of streets which he knew featured dropped curbs and no tactile paving. Isabel, a healthy volunteer with mobility limitations, walked in the road to avoid uneven and unstable pavements and noted that this unsatisfactory “solution” put her at risk of becoming involved in a road traffic accident. Cars parked on the pavement within his neighborhood meant that William, a healthy volunteer with mobility limitations, had to seek someone’s assistance if he wished to go out.

Several participants reported falls, some resulting in serious injury, and linked or attributed these to particular aspects of the environment. George, a participant with dementia, had experienced “a nasty fall” which his wife attributed to an uneven curb. He had “smashed (his) face up quite badly” as a result. Margaret, a healthy volunteer, had tripped on an uneven pavement, severely injuring her leg and was unable to leave the house for almost four weeks. Following the fall, her “confidence totally went (…) once an old person falls you have no idea what it’s like trying to get out”. Susan, a stroke survivor, had fallen several times and identified slippery, tiled surfaces as a cause. Several participants commented on the dangers of slippery and tiled surfaces and wondered why they were included in outdoor environments. Even participants who had not fallen reported fears of falling, “I’ve got to watch all the time because I’m so afraid that I’m going to fall or slip or trip” (Carole, healthy volunteer). Confirming past findings [26], falls, and a fear of falling, affected how some older adults used, perceived and interacted with the environment.

### 3.2. Role of Perceptions

For our participants, many assumptions, expectations and meanings were bound up or located within environmental details. At times, these seemed to inform an individual’s willingness to access and/or spend time within certain settings. The perceptions associated with street cameras and manicured areas of greenspace were particularly intriguing.

In terms of street-cameras, to some, this surveillance equipment suggested an unsafe environment. It was interpreted as a sign of crime and anti-social behavior, “what trouble are they expecting?” commented Isabel, a healthy volunteer, when discussing the inclusion of street cameras in residential areas. To others, street cameras implied threat and control with Alexander, a stroke survivor, noting, “(it would) make you feel like a prisoner if they were on your street”. For George, a participant with dementia, street cameras were simply “a nasty thing”. These various perceptions led some participants to feel uneasy occupying or accessing environments where street cameras were present. A much smaller number of participants believed it was sensible or beneficial to incorporate street cameras into environments, “they’ve got it made” noted Hazel, a healthy volunteer, when describing an area with such equipment.

Regarding manicured areas of greenspace, most participants interpreted such areas as unwelcoming, assuming that strict controls on permissible behavior would be enforced. Walking, sitting, picnicking or playing with grandchildren on neatly mown lawns were assumed to be prohibited. Comments such as: “when the grass is so well mown you feel you can’t go and walk on it, you’ll leave footprints” (Isabell, healthy volunteer), “I feel it’s somebody else’s territory and I’ve got to be careful” (Christine, healthy volunteer), “I think families wouldn’t be allowed (…) because it’s too pristine” (Linda, healthy volunteer) were common. For some, these perceptions and assumptions created a reluctance to visit and/or spend time within such spaces. Although rarely the case now, historically, parks and gardens within the UK have included fenced or protected lawns with signs warning visitors to “keep off the grass” [43]. We might assume that our older participants were socialized into these past norms and conventions. We might go on to speculate that these past principles of behavior appeared to continue to influence how our participants felt able to occupy and use these spaces.

### 3.3. Avoidance Behavior

Tied up with the role of perceptions, some participants felt increasingly vulnerable in certain temporal and spatial settings and, consequently, could actively eschew these situations. This avoidance behavior influenced where and when some individuals went out. Several participants reported a reluctance to go out in the evening or at night, or discussed changing the routes they took. Margaret, a healthy volunteer, extended her journey home to avoid using underpasses in the evening while Susan, a stroke survivor, avoided underpasses whenever possible: “anywhere there’s tunnels or underpasses I would just not go anyway near it if there was an alternative nearby”. Christine, a healthy volunteer, sometimes avoided using a rear entrance to access her block of flats in the evenings while, also in the evenings, Frances, a stroke survivor, would avoid being in places with lots of litter and which looked unkempt. Carole, a healthy volunteer, discussed her general reluctance to go out in the evenings and at night:

Carole: *Yeah, I’ll not go out at night. One, I can’t drive at night because I’ve got macular degeneration so I won’t drive when it’s dark and I don’t like, you know, walking from the bus stop to here, I’m not happy, I don’t feel secure in the dark*

Interviewer: *And is that something that has become more so as you’ve got older?*

Carole: *Yes, definitely. I mean, when I lived in the old house it’s away in a little backwater, nobody knows the two streets are there and it’s very dark and it didn’t bother me at all going out at night, but here I am in the main drag, and yet, I’m not happy at all going out.*

Like Carole, some participants reported perceiving hazard and risk in environments, and aspects of the environment, that had previously been of little or no concern. Joan, a healthy volunteer, now avoided walking along a canal towpath because she feared being knocked over by a cyclist. Several participants commented on the danger posed by cyclists riding on pavements and the shock experienced when a cyclist shoots past. Following her stroke, Janet discussed how she avoided crowded places as visual impairments and poor balance meant she felt particularly vulnerable in such spaces; she felt liable to fall or be knocked over and was concerned about being “on show”. Removed from such issues, a nearby beach had become a favourite place, almost a refuge: “there’s the beach and when I first had my stroke I could stagger and nobody looked at me (laughs) or I could fall and I didn’t hurt myself” (Janet, stroke survivor).

### 3.4. Environmental Aids

Various everyday aspects of the environment appeared to support, even encourage, outdoor mobility in more and less tangible ways. Seating provided resting points and so supported longer journeys. Paths were seen to signal, and support the possibility of, a journey. Local facilities provided a purpose and destination for trips out-of-the-home. Several stroke survivors and healthy volunteers suggested that stairs became easier to use when handrails and good lighting were present. Interactions between person-related and environmental factors were important in the context of environmental aids. For example, neuropathy, a condition resulting from damage to the nerves, meant that Caroline, a healthy volunteer with mobility limitations, was unable to walk very far or for very long. Within her neighborhood, benches provided a welcome place to rest enabling her to take longer walks:

“*Indeed, I do myself need to sit down if I go for a long walk, because of my neuropathy I can’t walk for very long without sitting down to recover, so yes it’s nice to have benches*”

Several stroke survivors commented on the need for benches, noting that after their stroke they became tired more easily and so needed to rest more often. Across our sample, toilets were considered important in supporting trips out of the home. Eric, a healthy volunteer, mentioned that their provision was particularly important for older people taking water tablets. Carole, a healthy volunteer, said she had felt forced to withdraw from a walking group because the routes selected did not always feature toilets and, due to a medical condition, walks without access to toilets would be impossible. Interestingly, while considered important, and while their closure by local government to save money was criticised, our participants rarely reported using public toilets: “I don’t think I’ve ever had to use one but I still think that they’re necessary” (Caroline, healthy volunteer). Instead, individuals sought out toilets in department stores. These were considered cleaner and more pleasant to use.

## 4. Discussion

Relatively similar experiences were reported across our diverse sample of participants in regards to environmental influences on walking. There was particular overlap between the experiences reported by the 15 healthy volunteers and the five stroke survivors. Pertinent here, and as previously noted, the P-E fit model [21] argues that interactions between person-related and environmental factors structure an individual’s experiences within an environment. Our “healthy” volunteers reported a range of diagnosed medical conditions and general health complaints, including impaired vision, joint and muscle pain, restricted movement, poor balance, heart conditions, fatigue and mobility problems. Some participants linked these complaints to the ageing process, “ageing is not for wimps” (Alexandra, healthy volunteer). The stroke survivors reported some similar complaints. (The participants with dementia volunteered no such information.) It might be, then, that overlaps in the health conditions and complaints reported by these two sets of participants explained why such similar experiences were identified. Across the two sets of participants there was, perhaps, some similarity in person-related factors.

Findings from this study confirm past research on the many ways in which aspects of the environment might influence walking in older adults. Various environmental barriers were identified and many correspond to the barriers reported in previous studies. Uneven pavements, tactile paving and curbs were, for example, found to complicate walking [13,44]. We found that some participants employed coping strategies to contend with these barriers. A number of environmental aids were also identified, such as benches, toilets and handrails and, again, these match aids reported elsewhere [13]. Linking to past research on the importance of street design to older people [13], typically the aids and barriers identified related to street-level, as opposed to neighborhood-level, items. Importantly, the study revealed that environmental details had the greatest impact on individuals reporting impairments.

Contributing new knowledge, the study has provided new insights into the role perceptions play in mobility choices. For our participants, located within many environments and environmental details were certain assumptions, expectations and meanings, and these could inform their walking behaviors. In these cases, an individual’s interpretation of an aspect of the environment, rather than the aspect per se, was key. It was not simply perceptions of safety that mattered, i.e., how safe an environment was perceived to be for walking, a concern identified in previous research [9]. As identified in the context of highly manicured areas of greenspace, whether an environment was perceived to be welcoming seemed, for instance, to count.

For built environment professionals, to support older adults to walk more, the findings suggest that attention ought to be directed towards providing and maintaining everyday (in a UK context) environmental details such as benches, pavements, street lighting, handrails and dropped curbs. We can see such concerns already reflected in some built environment guidance. Examples include the UK’s Manual for Streets (2007) [45] and Manual for Streets 2—Wider Application of the Principles (2010) [46]. For health and aligned professions wishing to encourage walking in the older population, our findings suggest that one approach might be to plot, and provide maps of, accessible-to-all walking routes which avoid “problem” items within the environment. Relevant here, within the UK, Walk Unlimited have created multiple “Walk4Life” route maps which highlight paths that avoid steps and steep gradients [47]. Walking initiatives targeted at this population could focus on such routes. Alternatively, they could focus on indoor walking in modern shopping centres, a practice termed “mall walking” in America [48], where access, benches and toilets can be (somewhat) assured.

Limitations of the study include use of a small, unrepresentative sample which makes extrapolating to wider populations difficult. Particular difficulties were encountered when seeking to recruit stroke survivors and people with dementia. Future research could adopt alternative recruitment strategies such as, where appropriate, engaging with peer-support groups—see a study by Brookfield and Mead (2016) where participants with experience of stroke were successfully recruited through stroke clubs [25].

## 5. Conclusions

Interview and focus group data collected from 22 seniors aged 60 years and over, presenting a range of functional and cognitive impairments, highlighted the uncommon impact of common environmental details on walking in assorted older adults. Everyday items such as pavements, curbs, street lighting, benches and handrails were found to complicate or support walking. In some instances, the meanings and possibilities that older adults located within aspects of the environment were what mattered. Interactions between person-related and environmental factors appeared key in structuring older adults’ walking experiences and behaviors. For built environment professions, the findings suggest that to support walking in this population commonplace environmental details ought to attract attention with decisions on their look, location and preponderance taking into account the meanings and possibilities that they can hold for older people. Further, for health and aligned professionals wishing to encourage walking in this demographic, accessible-to-all walking routes which avoid problematic environmental details could be produced and promoted.
